# The effect of estrogen on prolidase-dependent regulation of HIF-1α expression in breast cancer cells

**DOI:** 10.1007/s11010-013-1623-9

**Published:** 2013-04-03

**Authors:** Arkadiusz Surazynski, Wojciech Miltyk, Izabela Prokop, Jerzy Palka

**Affiliations:** 1 Department of Medicinal Chemistry, Medical University of Bialystok, Kilińskiego 1, 15-089 Bialystok, Poland; 2 Department of Pharmaceutical Analysis, Medical University of Bialystok, Kilińskiego 1, 15-089 Bialystok, Poland

**Keywords:** Estrogen, HIF-1α, MCF-7, MDA-MB-231, Prolidase

## Abstract

The role of estrogen in breast cancer progression and activation of prolidase activity and HIF-1α led us to study the effect of estrogen on nuclear HIF-1α expression in breast cancer estrogen-dependent MCF-7 and estrogen-independent MDA-MB-231 cells. We have found that in MCF-7 cells (expressing α and β estrogen receptor) cultured without estrogen receptor activator (phenol red, estradiol), HIF-1α was down-regulated, compared to the cells cultured with estrogen receptor activator. This effect was not observed in MDA-MB-231 cells (expressing only β estrogen receptor), suggesting that α estrogen receptor is involved in down-regulation of HIF-1α. However, in MDA-MB-231 cells (expressing high prolidase activity) cultured in the presence of prolidase substrates, Gly-Pro or Gly-HyPro, HIF-1α expression was induced in a dose-dependent manner, independently of estrogen receptor activation. In MCF-7 cells (with constitutively low prolidase activity) the effect of studied iminodipeptides on HIF-1α expression was much less pronounced but it was estrogen-dependent, showing importance of prolidase activity in mechanism of this process. The data were supported by confocal microscopy bio-imaging of HIF-1α in nucleus of MCF-7 and MDA-MB-231 cells that were cultured in the presence and absence of estrogen activator and prolidase substrates. It suggests that estrogen receptor may represent important therapeutic target in pharmacotherapy of estrogen receptor positive breast cancer, while ECM degradation enzymes, including prolidase may represent target in pharmacotherapy of estrogen receptor negative breast cancers.

## Introduction

The role of estrogen in the promotion and development of breast cancer is well documented by epidemiological data and the therapeutic efficacy of anti-estrogen therapy [1]. More direct evidence was obtained with estrogen receptor (ER) positive breast cancer cell lines in which estrogens were found to stimulate the proliferation of these cells both in culture [2] and in nude mice [3]. However, ER positive tumor cells are poorly metastatic compared to ER negative ones [4] and more responsive to antiestrogens [5]. It may suggest a regulatory role of estrogens in breast cancer cell metastasis.

Estrogens are implicated in collagen metabolism and cell growth in several cell types [6–8]. Cell locomotion requires extensive degradation of extracellular matrix (ECM) components, including collagens [9]. Although extracellular metalloproteinases initiate the breakdown of collagen, the final step of its degradation is mediated by cytoplasmic prolidase.

Prolidase (E.C.3.4.13.9) is the enzyme that catalyzes the final step in ECM degradation by releasing proline or hydroxyproline from the carboxyl terminus of imidodipeptides [10]. Our previous study showed that prolidase participates not only in post-transcriptional regulation of collagen biosynthesis but also is involved in regulation at transcriptional level [11]. Several reports suggest that prolidase through regulation of expression of growth factors (e.g., vascular endothelial growth factor, VEGF; transforming growth factor β, TGF-β) and transcription factors (e.g., hypoxia inducible factor 1α, HIF-1α) may play important role in wound healing, inflammation, and angiogenesis [11–14].

The most representative study demonstrated the role of products of prolidase activity, proline or hydroxyproline in regulation of HIF-1α degradation. Over-expression of prolidase resulted in increased HIF-1α levels and elevated expression of HIF-1α-dependent gene products, VEGF, and glucose transporter-1 (Glut-1). Mechanism for the accumulation of HIF-1α was due to the inhibition of von Hippel-Lindau (VHL)-dependent degradation [11].

The current study was therefore undertaken to characterize the effect of estrogen on HIF-1α expression in breast cancer estrogen-dependent MCF-7 and estrogen-independent MDA-MB-231 cell lines.

## Materials

l-Glycyl-l-proline, l-proline, and Dulbecco’s modified Eagle’s medium with or without phenol red (DMEM) or controlled process serum replacement I, (CPSR1), sodium bicarbonate, penicillin, streptomycin, fetal bovine serum (FBS), Dulbecco’s phosphate-buffered saline (DPBS), 5-bromo-4-chloro-3-indolyl phosphate/nitro blue tetrazolium liquid substrate reagent (BCIP/NBT), Monoclonal (mouse) anti-β-actin antibody, anti-Mouse IgG AP antibody, were purchased from Sigma Chemicals Co., USA, as were most other chemicals used. Nuclear extracts NE-PER kit was purchased from Pierce (Rockford, IL, USA). Monoclonal (mouse) anti-estrogen receptor α (ER α), was obtained from Santa Cruz Biotechnology, Inc., USA, monoclonal (mouse) anti-HIF-1α (WB) was obtained from BD Transduction Laboratories, CA, USA, monoclonal (mouse) anti-Hypoxia Inducible Factor 1 Alpha (HIF-1alpha) (IF) StressMarq from Biosciences Inc. Canada, Goat Polyclonal anti-Mouse IgG-heavy and light chain antibody conjugate Fluorescein Isothiocyanate (FITC), from Bethyl Laboratories, Inc. USA. Nitrocellulose membrane (0.2 μm), sodium dodecylsulphate (SDS), polyacrylamide, molecular weight standards, and Coomassie Brilliant Blue R-250 were received from Bio-Rad Laboratories USA.

### Cell cultures

The studies were performed on estrogen-dependent MCF-7 cells, expressing α and β receptor and on estrogen-independent MDA-MB-231 cells, expressing only β receptor. MCF-7 and MDA-MB-231 cells were maintained in DMEM without phenol red supplemented with 10 % CPSR1, 50 U/ml penicillin, 50 μg/ml streptomycin at 37 °C in a 5 % CO_2_ incubator. Cells were cultured in Costar flasks or in BD Falcon™ 96-well black/clear bottom tissue culture plates (optimized for imaging applications). For analysis, except bioimaging, sub-confluent cells were detached with 0.05 % trypsin, 0.02 % EDTA in calcium-free phosphate-buffered saline, counted in hemocytometer and plated at 5 × 10^5^ cells per well of six-well plates (Nunc) in 2 ml of growth medium. Cells reached about 80 % of confluence at day 2 after plating and in most cases such cells were used for the assays.

### Western blot analysis

Nuclear and cytoplasmic extracts were prepared by the standard protocol described in the NE-PER kit from Pierce, (Rockford, IL, USA). For Western blots, equal amounts of cell extract proteins (cytoplasmic or nuclear) were electrophoresed on SDS-PAGE. Slab SDS/PAGE was used, according to the method of Laemmli [15]. After SDS-PAGE, the gels were allowed to equilibrate for 5 min in 25 mM Tris, 0.2 M glycine in 20 % (v/v) methanol. The proteins were transferred to 0.2 μm pore-sized nitrocellulose at 100 mA for 1 h by using a LKB 2117 Multiphor II electrophoresis unit. The nitrocellulose was incubated with: monoclonal anti-HIF-1α and ER α, at concentration 1:1,000 in 5 % dried milk in Tris-buffered saline with Tween 20 (TBS-T) (20 mmol/l Tris–HCl buffer, pH 7.4, containing 150 mmol/l NaCl and 0.05 % Tween 20) for 1 h. In order to analyze HIF-1α and ER α second antibody-alkaline phosphatase conjugated, anti-Mouse IgG (whole molecule) was added at concentration 1:5,000 in TBS-T and incubated for 30 min slowly shaking. Then nitrocellulose was washed with TBS-T (5 × 5 min) and submitted to 5-bromo-4-chloro-3-indolyl phosphate/nitro blue tetrazolium liquid substrate reagent (BCIP/NBT).

### Prolidase activity

The activity of prolidase was determined according to the method of Myara [10], which is based on colorimetric determination of proline using Chinard’s reagent. Cells were scraped off and centrifuged at 200×*g* for 15 min and the supernatant was discarded. The cell pellet was suspended in 1 ml of 50 mM HEPES, pH 7.8, and sonicated for 3 × 10 s at 0 °C. Samples were then centrifuged (12,000×*g*, 30 min) at 4 °C and the supernatant was used for protein determination (Bradford method). Activation of prolidase requires incubation with Mn(II): 100 μl of cell extract supernatant was mixed with 100 μl of 50 mM HEPES, pH 7.8 containing MnCl_2_ at a final concentration of 1 mM in the mixture. After incubation for 24 h at 37 °C, the prolidase reaction was initiated by adding 100 μl of the activated mixture to 100 μl of 94 mM glycyl-proline (Gly-Pro) for a final concentration of 47 mM. After additional incubation for 1 h at 37 °C, the reaction was terminated with the addition of 1 ml of 0.45 M trichloroacetic acid. To parallel blank tubes, trichloroacetic acid was added at time “zero”. Samples were centrifuged at 10,000×*g* for 15 min. The released proline was determined by adding 0.5 ml of the trichloroacetic acid supernatant to 2 ml of a 1:1 mixture of glacial acetic acid: Chinard’s reagent (25 g of ninhydrin dissolved at 70 °C in 600 ml of glacial acetic acid and 400 ml of 6 M orthophosphoric acid) and incubated for 10 min at 90 °C. The amount of proline released was determined colorimetrically by monitoring absorbance at 515 nm and calculated using proline standards. Enzyme activity was reported in nanomoles of proline released per minute per milligram of protein.

### Immunofluorescence

Cells were cultured in BD Falcon™ 96-well black/clear bottom tissue culture plates optimized for imaging applications at 10,000 cells per well. After incubation, cells were rinsed with PBS and fixed with 3.7 % formaldehyde solution at room temperature for 10 min. After fixation, cells were washed three times with PBS and permeabilized with 0.1 % Triton X-100 solution at room temperature for 5 min. Then, cells were washed twice with PBS, and non-specific binding was blocked by addition of a 3 % FBS solution and incubated at room temperature for 30 min. After that time the cells were rinsed, incubated with anti-HIF-1α mouse monoclonal antibody for 1 h at room temperature, washed three times with PBS and incubated with fluorescent (FITC) anti-mouse secondary antibody for 60 min in the dark. After washing, nuclei were stained with Hoechst 33342 (2 μg/ml) and analyzed using confocal microscopy imaging.

### Confocal microscopy

Cells were imaged with a BD Pathway 855 confocal system using a 20× (0.75 NA) objective. Cell populations were analyzed for cytoplasmic fluorescence intensity. Images of FITC-labeled cells were acquired using a 488/10 excitation laser and a 515LP emission laser.

### Statistical analysis

In experiments the mean values for six assays ± standard deviations (SD) were calculated. The results were analyzed using the ANOVA method, accepting *P* < 0.01, as significant.

## Results

**Fig. 1:**
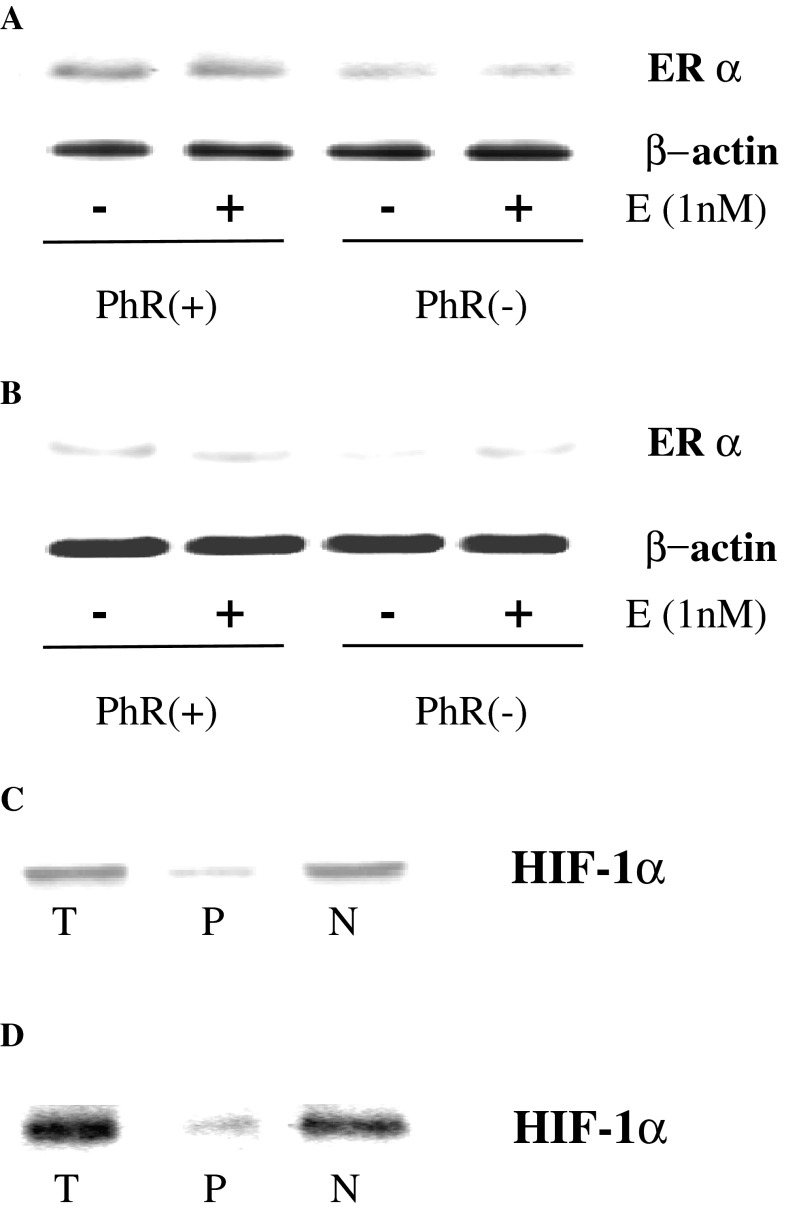
Western blot for estrogen receptor α (ERα) and β-actin in MCF-7 (**a**) and MDA-MB-231 (**b**) cells cultured in the absence (−) or presence (+) of 1 nM estrogen (E) in DMEM with (PhR+) or without (PhR−) phenol red and 10 % CPSR1 for 24 h. Western blot for HIF-1α in MCF-7 (**c**) and MDA-MB-231 (**d**) cells in total (T), cytoplasmic (P), or nuclear (N) extracts

The studies were performed on estrogen-dependent MCF-7 cells, expressing α and β estrogen receptor and on estrogen-independent, MDA-MB-231 cells, expressing only β estrogen receptor. In cultured cells, phenol red contained in medium mimics activity of estrogens [16]. In order to test the effect of estradiol on estrogen receptor α expression in MCF-7 and MDA-MB-231 cells, they were treated for 24 h with or without 1 nM estradiol in medium with or without phenol red (PhR), containing 10 % CPSR 1. We found that in MCF-7 cell incubated without phenol red in medium, expression of estrogen receptor α (ER α) was markedly reduced compared to cells cultured in medium with phenol red (Fig. 1a). An addition of 1 nM estradiol to cells cultured in phenol red slightly up-regulated ER α expression while had no effect on the receptor expression in cells cultured without phenol red (Fig 1a). In MDA-MB-231 cells the presence or absence of estradiol in medium with or without phenol red had no effect on expression of estrogen receptor α (Fig. 1b).

**Fig. 2:**
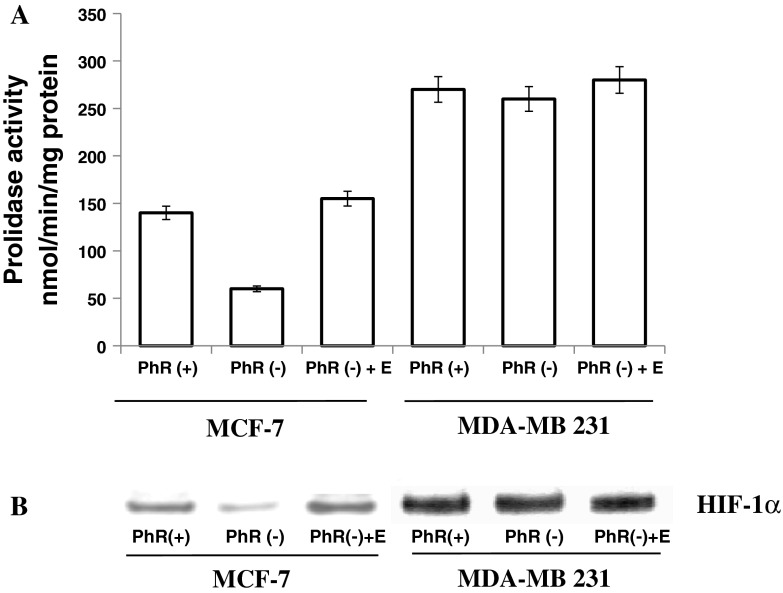
Prolidase activity (**a**) and expression of HIF-1α (**b**) in MCF-7 and MDA-MB-231 cells, cultured in DMEM with PhR(+), without PhR(−) or without PhR(−) phenol red in the presence of 1 nM estrogen (+E) and 10 % CPSR1 for 24 h. Data shown represent mean ± SD of 3 determinations and the difference is statistically significant by the ANOVA method *P* < 0.01

Estrogens were found to up-regulate prolidase activity [8, 17]. In order to evaluate the effect of estrogen on prolidase activity, the studied cells were cultured with (+) or without (−) PhR and 1nM estradiol. We found that in the absence of estrogen in medium (PhR−), prolidase activity in MCF-7 cells was markedly reduced (by about 60 %), compared to the cells cultured in medium with PhR. An addition of 1 nM estradiol to the medium without PhR restored the enzyme activity to the level found in cells cultured with PhR (Fig 2a). In case of estrogen-independent MDA-MB-231 cells (Fig. 2a) there was no effect of PhR or estradiol on prolidase activity.

Since prolidase activity is involved in regulation of HIF-1α, we decided to evaluate the expression of this transcription factor in studied cells in the same conditions as those described for prolidase activity assay. We have found that the expression of HIF-1α in both cell lines reflects prolidase activity (Fig. 2a, b). In MCF-7 cells, cultured in the absence of estrogen receptor activator, a decrease in HIF-1α expression was found, compared to the cells cultured with estrogen receptor activator. This effect was not observed in estrogen-independent MDA-MB-231 cells.

**Fig. 3:**
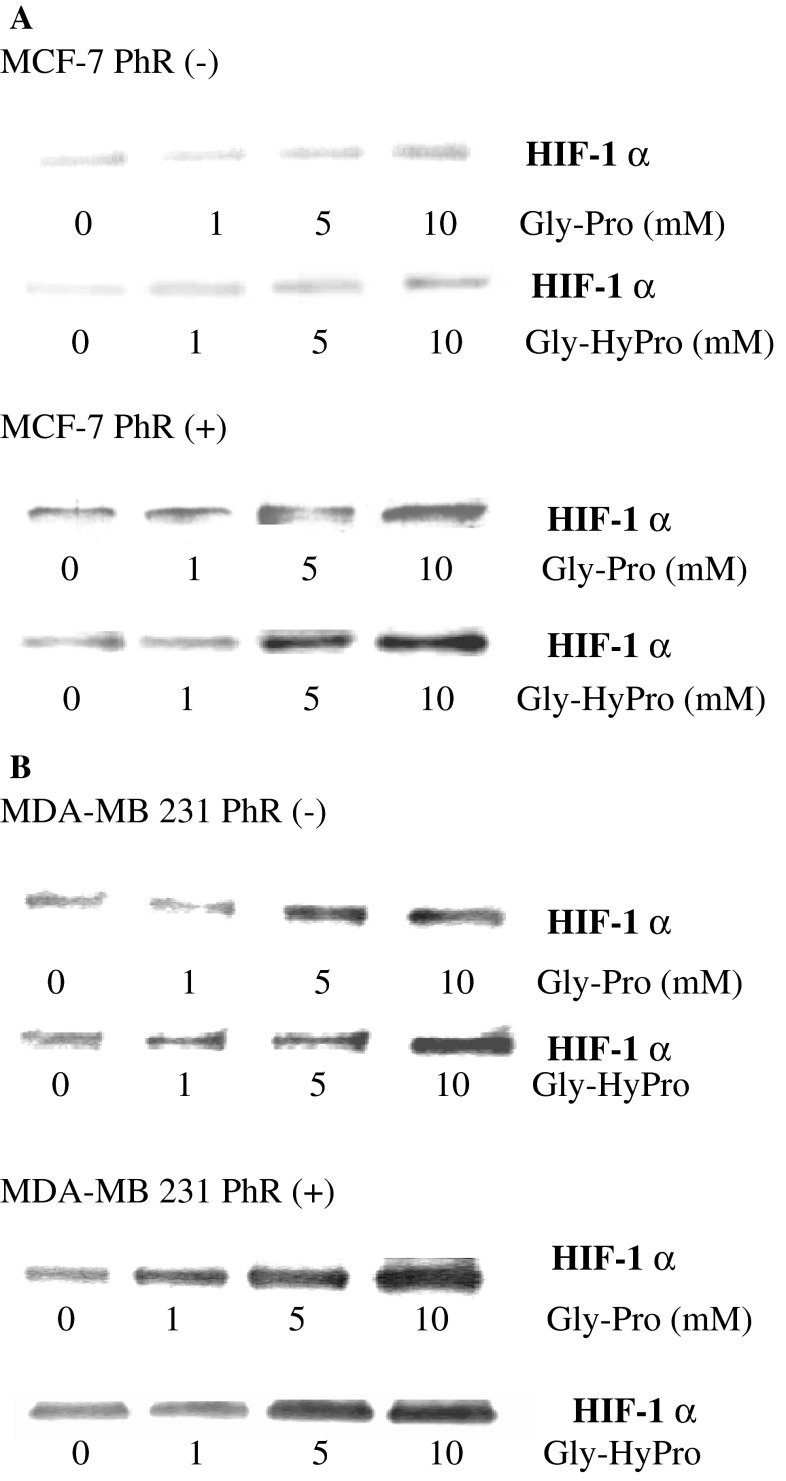
Western blot analysis for HIF-1α in MCF-7 (**a**) and MDA-MB-231 (**b**) cells cultured in the absence (−) or presence (+) of phenol red in DMEM with 10 % CPSR1 and submitted for 24 h to different concentrations of Gly-Pro or Gly-HyPro. Samples used for electrophoresis consisted of 20 μg of protein of pooled cell extracts (*n* = 6)

**Fig. 4:**
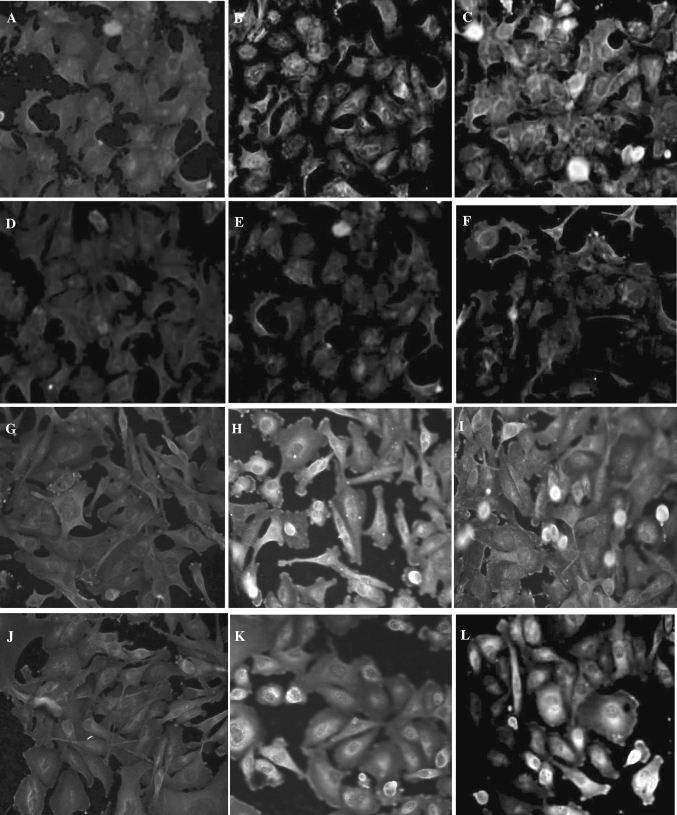
Imaging analysis for HIF-1α in MCF-7 cells cultured in the presence (+) of phenol red in DMEM with 10 % CPSR1 (**a**) and submitted for 24 h to 10 mM concentration of Gly-Pro (**b**) or Gly-HyPro (**c**) and MCF-7 cells cultured in the absence (−) of phenol red in DMEM with 10 % CPSR1 (**d**) and submitted for 24 h to 10 mM concentration of Gly-Pro (**e**) or Gly-HyPro (**f**). MDA-MB-231 cells cultured in the presence (+) of phenol red in DMEM with 10 % CPSR1 (**g**) and submitted for 24 h to 10 mM concentration of Gly-Pro (**h**) or Gly-HyPro (**i**) and MDA-MB-231 cells cultured in the absence (−) of phenol red in DMEM with 10 % CPSR1(**j**) and submitted for 24 h to 10 mM concentration of Gly-Pro (**k**) or Gly-HyPro (**l**)

In order to establish whether products of prolidase activity affect HIF-1α expression we exposed cells to different concentrations of prolidase substrates, i.e., Gly-Pro or Gly-HyPro and monitored expression of HIF-1α. In MDA-MB-231 cells cultured with Gly-Pro or Gly-HyPro, the expression of HIF-1α increased accordingly to the increased concentration of imidodipeptides, independently of the estrogen receptor activation (Fig. 3b). However, in MCF-7 cells cultured without estrogen, the increase in expression of HIF-1α due to imidodipeptides is markedly lower then in the cells cultured in medium with estrogen (Fig. 3a). These results show that the effect of estrogen on the levels of HIF-1α in nuclear extracts is related to prolidase function. The data are supported by confocal microscopy bio-imaging of HIF-1α in nucleus of MCF-7 and MDA-MB-231 cells that were cultured in the presence and absence of estrogen activator and prolidase substrates (Fig. 4).

## Discussion

Estrogens are known to stimulate the growth of normal and transformed epithelial cells [18]. The mechanism of their action involves interaction with estrogen receptor that after binding of ligand is targeted to the nucleus as an transcription factor [19]. However, activation of estrogen receptor is regulated not only by its ligands but also by number of factors, including kinases, phosphatases, and growth factors [20]. For instance, in endometrial cancer cells p38 mitogen-activated protein kinase (MAPK) signaling phosphorylates estrogen receptor α, promoting its nuclear localization [21].

Despite the fact that estrogens play an important role in the promotion and development of female epithelial-derived cancer [22], it has been postulated that estrogen receptor (ER) positive cancer cells are poorly metastatic compared to ER negative ones [4]. The mechanism of the regulatory role of estrogens in cancer cell growth and metastasis, however, is not understood. Although in this report we do not study growth and metastasis of breast cancer cells, the approach to understand the effect of estrogens on the processes is focused on the regulation of HIF-1α expression.

It seems that in the context of breast cancer, the most important is contribution of prolidase to regulation of HIF-1α. The stability and activity of HIF-1α are regulated by various post-translational modifications, hydroxylation, acetylation, and phosphorylation. Under normoxia, the HIF-1α subunit is rapidly degraded via the von Hippel-Lindau tumor suppressor gene product (pVHL)-mediated ubiquitin–proteasome pathway. The association of pVHL and HIF-1α under normoxic conditions is triggered by the hydroxylation of prolines and the acetylation of lysine within a polypeptide segment known as the oxygen-dependent degradation (ODD) domain. On the contrary, in the hypoxia condition, HIF-1α subunit becomes stable and interacts with coactivators such as p300/CBP to modulate its transcriptional activity. Overexpression of prolidase resulted in increased nuclear HIF-1α levels and elevated expression of HIF-1-dependent gene products, vascular endothelial growth factor (VEGF), and glucose transporter-1 (Glut-1). The activation of HIF-1α-dependent transcription was shown by prolidase-dependent activation of HRE-luciferase expression. In previous studies we used an oxygen-dependent degradation domain (ODD)-luciferase reporter construct as a surrogate for HIF-1α in an in situ prolyl-hydroxylase assay. Since this reporter is degraded by VHL-dependent mechanisms, increased levels of HIF-1α with prolidase expression were due to decreased hydroxylation. Additionally, the differential expression of prolidase in two breast cancer cell lines MCF-7 and MDA-MB-231 showed prolidase-dependent differences in HIF-1α levels [11]. These findings show that metabolism of imidodipeptides by prolidase plays a previously unrecognized role in angiogenic signaling, cell proliferation/survival, and glucose metabolism.

**Scheme 1:**
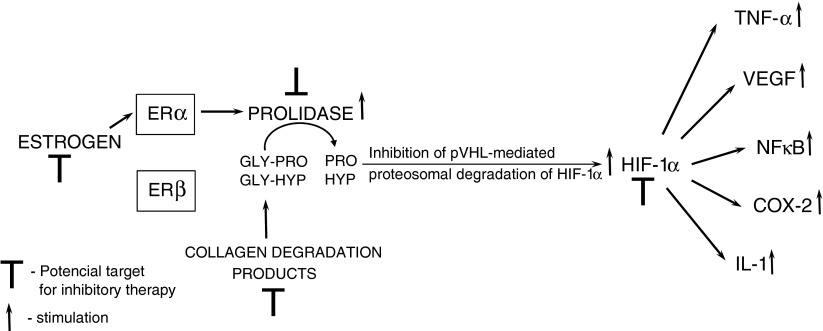
.

In this study we have found that prolidase activity reflects nuclear localization of HIF-1α in breast cancer cells. Furthermore, we have found that this phenomenon is due to estrogen receptor α subunit. The evidence was presented that up-regulation of prolidase activity by estrogen take place only in MCF-7 cells, expressing α estrogen receptor and not in MDA-MB-231 cells lacking this receptor. Therefore, we suggest that α estrogen receptor may be crucial for estrogen-dependent up-regulation of prolidase activity. Increase in prolidase activity in turn accelerate release of proline and hydroxyproline from iminodiopeotides, that inhibit degradation of HIF-1α, contributing to increase in nuclear localization of this transcription factor. In MCF-7 cells this mechanism contribute to activation of HIF-1α function. However, in MDA-MB-231 cells with constitutively high prolidase activity, the HIF-1α expression is independent of estrogen receptor activation. It seems that in MDA-MB-231 cells, HIF-1α expression is regulated by final products of extracellular matrix (ECM) degradation, iminodipeptides, that are substrate for prolidase. The data presented in this study documented higher effectiveness of iminodipeptides in up-regulation of HIF-1α in MDA-MB-231, than in MCF-7 cells. It suggests that ECM integrity is of critical importance for the maintenance of normal tissue. The linkage of ECM and hypoxia suggests that the metabolic system senses ECM degradation as a stress condition that requires neoangiogenesis. In fact, both HIF-1α and prolidase are involved in angiogenesis. Patients with prolidase deficiency exhibit defective wound healing, resulting in extensive skin ulcerations and immunodeficiency that contribute to frequent infections. Of special interest, histologic features on postmortem examination included marked angiopathy not only in skin ulcerations [23] but also in internal tissues [24], suggesting that the deficiency in prolidase may cause defective angiogenesis. Taking together the data suggest that estrogen receptor may represent important therapeutic target in pharmacotherapy of ER positive breast cancer, while ECM degradation enzymes, including prolidase may represent target in pharmacotherapy of ER negative breast cancers. The potential mechanism of this process is outlined in Scheme 1.
